# Clinical value of the venous volume excess ultrasound score (VExUS) for volume management following cardiopulmonary bypass (CPB)

**DOI:** 10.1007/s40477-026-01200-5

**Published:** 2026-08-07

**Authors:** Lijun Guo, Wei Gong, Huiyan Zhang, Hongli Gu, Qiong Li

**Affiliations:** 1 Department of Neurology, Affiliated Hospital of Xiangnan University, Chenzhou, 423000 Hunan China; 2 Department of Critical Care Medicine, The First People’s Hospital of Chenzhou, 102 Luo Jia Jin Street, Chenzhou, 423000 China; 3 Department of Ultrasound, The First People’s Hospital of Chenzhou, 102 Luo Jia Jin Street, Chenzhou, 423000 China; 4 Emergency Department, The First People’s Hospital of Chenzhou, Chenzhou, 423000 China

**Keywords:** VExUS, CPB, Postoperative, Volume management

## Abstract

**Objective:**

To assess the clinical value of VExUS-guided volume management in patients following CPB surgery.

**Methods:**

A single-center, prospective, randomized controlled trial was performed. All adult patients undergoing CPB surgery between January 2024 and October 2025 were consecutively enrolled and randomly allocated in a 1:1 ratio to either a VExUS-guided intervention group or a conventional control group. Baseline demographics, intraoperative surgical parameters, serial postoperative fluid and transfusion metrics, and clinical outcomes were compared between the two cohorts. Intergroup comparisons were conducted using two-tailed statistical tests, with a significance level of *P* < 0.05.

**Results:**

A total of 89 eligible participants completed the study, including 46 in the VExUS group and 43 in the control group. Baseline clinical profiles and all intraoperative variables showed no statistically significant intergroup differences (all *P* > 0.05). At 12 h and 24 h postoperatively, the median albumin transfusion volume was 0 g in both groups; however, the VExUS-guided cohort exhibited broader data distribution and a larger proportion of patients receiving high-dose albumin administration, with significant between-group differences (*P* = 0.007 and* P* = 0.015, respectively). The median postoperative hospital stay was markedly shorter in the VExUS group (15 days vs. 19 days,* P* = 0.027). No significant differences were observed in intensive care unit (ICU) or total mechanical ventilation time. Although the cumulative net fluid balance did not reach statistical significance between groups, the VExUS-guided group demonstrated a more prominent negative fluid balance trend at 48 h postoperatively, reflecting greater systemic decongestion.

**Conclusion:**

VExUS-directed volume management optimizes personalized fluid therapy and shortens postoperative hospital stay in patients undergoing CPB surgery.

## Introduction

Fluid resuscitation and targeted volume regulation constitute cornerstones of critical care, especially for patients with unstable hemodynamics [1]. Substantial clinical evidence has confirmed that individualized fluid therapy during resuscitation substantially improves survival and organ function in critically ill patients. Perioperative volume monitoring remains a persistent clinical challenge for patients following CPB surgery. Comprehensive volumetric assessment requires integrated evaluation of global pathophysiological status, cardiac structural and functional performance, and ventricular compliance, alongside consideration of CPB-induced systemic vasodilation and fluid redistribution during postoperative rewarming [2, 3 ].

Volume overload is a prevalent iatrogenic complication following CPB, predominantly driven by systemic venous congestion secondary to fluid excess and impaired cardiac output. Elevated capillary hydrostatic pressure resulting from widespread venous congestion disrupts vital organ microcirculation and elevates the risk of postoperative complications [4, 5 ]. Traditional hemodynamic monitors have inherent limitations. Central venous pressure (CVP) measurements are heavily confounded by intrathoracic pressure and chest wall compliance, The 2024 clinical practice guideline issued by the ESICM discourages sole reliance on central venous pressure (CVP) to direct fluid resuscitation [6]. Pulse contour cardiac output (PiCCO) generates detailed hemodynamic data but requires invasive vascular access and cannot directly quantify congestion within visceral venous beds. These constraints prevent clinicians from designing precise, patient-tailored fluid regimens [7].

VExUS is a novel noninvasive tool that quantifies systemic venous congestion. Drawing on the landmark investigation by Beaubien-Souligny [8].This bedside assessment protocol comprises four sequential steps. First, the inferior vena cava (IVC) diameter is measured. For IVC diameters ≥ 2 cm, Doppler waveform analysis of the hepatic, portal, and intrarenal veins is subsequently performed. Venous congestion severity is then stratified into grades 0 through 3 according to characteristic waveform derangements—such as hepatic vein systolic reflux or portal vein pulsatility exceeding 50%. By integrating IVC diameter measurements and Doppler flow waveforms from the hepatic vein, portal vein, and renal interlobular veins, enabling direct evaluation of impaired visceral venous return [9].The technique offers distinct advantages: bedside operability, non-invasiveness, capacity for serial dynamic monitoring, and standardized quantitative data. This prospective trial aimed to evaluate the clinical efficacy of VExUS-guided volume management in post-CPB patients to generate evidence supporting standardized and refined postoperative fluid therapy protocols.

## Methods

### Study design and participants

This single-center, prospective, randomized controlled trial recruited all adult patients undergoing elective or emergency CPB surgery between January 2024 and October 2025 at The First People’s Hospital of Chenzhou.

### Inclusion criteria

① Age ≥ 18 years; ② Signed written informed consent from patients or legal guardians, and agreement to follow-up.

### Exclusion criteria

① Pregnant or lactating women; ② Intra-abdominal pressure (IAP > 12 mmHg, measured via bladder manometry) or significant bowel distension affecting ultrasound imaging; ③ Cirrhosis of the liver, renal artery stenosis resulting in chronic kidney disease (CKD stage ≥ 3), constrictive pericarditis, acute pulmonary embolism, or hyperdynamic cardiac state; ④ ECMO support; ⑤ Persistent atrial fibrillation (heart rate > 100 beats/minute).

The trial protocol strictly complied with the principles outlined in the Declaration of Helsinki and received institutional ethics committee approval (Approval Number: 2025069). Written informed consent was obtained from all patients or their legal guardians prior to enrollment. The trial was pre-registered on ClinicalTrials.gov (Registration Number: CTR2600118336). A 1:1 randomization sequence was generated using SPSS 26.0. Ultrasound operators only completed image collection and VExUS scoring without participating in clinical treatment decisions; attending physicians carried out treatment according to group assignment; All statistical analysts remained blinded to group assignment to mitigate detection bias.

### Surgical and perioperative management

All patients underwent transthoracic echocardiography preoperatively to evaluate baseline cardiac function. Operations were performed under total intravenous anesthesia combined with mild hypothermia. Intraoperatively, right internal jugular venous cannulation was performed for vasoactive drug infusion and fluid delivery, while radial arterial cannulation allowed continuous invasive arterial pressure monitoring and blood sampling. All surgical procedures utilized median sternotomy, including coronary artery bypass grafting, cardiac valve replacement, and aortic arch reconstruction.

### Standard resuscitation targets

Heart rate (HR): Adults: 60–100 beats per minute (bpm); Arterial blood pressure (ABP): Systolic arterial pressure (SAP): 90–139 mmHg; Diastolic arterial pressure (DAP): 60–89 mmHg; Mean arterial pressure (MAP): ≥ 65 mmHg (critical care standard perfusion threshold);Urine output: ≥ 0.5 mL/(kg·h); Blood lactate: Normal range 0.5–1.6 mmol/L; resuscitation target ≤ 2.0 mmol/L, Central venous oxygen saturation (ScvO_2_): 70%–80%; Arteriovenous carbon dioxide partial pressure difference (P(v-a)CO_2_): < 6 mmHg.

### Conventional control group

Patients received conventional volume management based on routine clinical and laboratory parameters. Attending physician comprehensively assessed volumetric status based on heart rate, arterial blood pressure, urine output, serum lactate, ScvO_2_, and P(v-a)CO_2_, with fluid regimens dynamically adjusted at the clinician's discretion. Consistent serial measurements of IVC diameter, left ventricular outflow tract stroke volume, and continuous CVP monitoring were not routinely documented per institutional standard workflows for this cohort. The primary therapeutic endpoint was sustained hemodynamic stability and adequate end-organ perfusion.

### VExUS-guided group

In addition to conventional monitoring and interventions applied to the control group, standardized serial VExUS ultrasound assessments and targeted fluid adjustments were integrated into clinical care. All VExUS scans were performed by two intensivists with ≥ 5 years of critical care ultrasound experience using a Mindray M9 portable color Doppler ultrasound system equipped with a 5-MHz convex probe. Scheduled monitoring time points included immediately upon ICU admission postoperatively, 12 h postoperatively, and 24 h postoperatively. For each ultrasonographic assessment, patients were positioned in the supine or lateral position with full abdominal exposure. Expiratory IVC diameter was measured at a distance of 2 cm proximal to the right atrial junction, with the average of three measurements recorded. Doppler spectral tracings of the hepatic vein, main portal vein, and renal interlobular veins were sequentially acquired, and VExUS grades were calculated per standardized grading criteria. When the VExUS score exceeded 1 (consistent with significant systemic venous congestion), a predefined negative fluid balance protocol was activated, including optimized optimized loop diuretic administration and crystalloid infusion restriction. Tissue perfusion markers were continuously monitored throughout the intervention to avoid end-organ hypoperfusion.

### Data collection

#### Baseline variables

Demographic characteristics, chronic comorbidities, admission vital signs, baseline laboratory biomarkers, and vasoactive-inotropic score (VIS).

#### Intraoperative variables

Total surgical duration, CPB circulatory support time, aortic cross-clamp duration, intraoperative APACHE II score, and cumulative intraoperative fluid balance.

#### Postoperative fluid and transfusion parameters

Cumulative fluid intake, total fluid output, and net fluid balance at 12 h, 24 h, and 48 h postoperatively; transfusion volumes of packed red blood cells, fresh frozen plasma, and human albumin; serial hemodynamic and laboratory data. The VExUS group also recorded expiratory IVC diameter, venous Doppler waveforms, and global VExUS grade at each predefined time point.

#### Clinical endpoints

**Primary outcome:** postoperative hospital length of stay; **secondary outcomes:** ICU length of stay, total mechanical ventilation time, incidence of renal replacement therapy during hospitalization, and discharge rehabilitation status.

### Statistical analysis

Statistical analyses were performed using R software version 4.5.0. Normality testing of continuous variables was conducted using the Shapiro–Wilk test. Normally distributed continuous data were reported as mean ± standard deviation and compared using independent-samples t-tests. Non-normally distributed continuous variables were reported as median (interquartile range [IQR]; P25–P75), with intergroup comparisons performed using the Mann–Whitney U test. Categorical variables were summarized as n (%) and analyzed using the chi-squared test or Fisher’s exact test for small expected frequencies. All statistical hypothesis tests were two-tailed, with the threshold for statistical significance set at *P* < 0.05.

## Results

### Baseline patient clinical characteristics

A total of 89 patients were ultimately enrolled, including 49 males (55.06%) and 40 females (44.94%), with a median age of 60 years and a median BMI of 22.86 kg/m^2^. Patients were randomly assigned into the VExUS-guided group (*n* = 46) and the control group (*n* = 43). Three patients in the control group were excluded due to incomplete data and unqualified ultrasound images, without affecting the original randomization.

The most prevalent chronic comorbidities across the full cohort were hypertension (40 patients, 44.94%), type 2 diabetes mellitus (13 patients, 14.61%), and coronary artery disease (9 patients, 10.11%). No statistically significant intergroup differences were detected in demographic profiles, comorbidity distribution, admission vital signs, or baseline laboratory biomarkers (all* P* > 0.05), confirming the balanced baseline characteristics between study arms (Table 1).

**Table 1:** Baseline characteristics of enrolled patients

Characteristics	VExUS (*n* = 46)	Control (*n* = 43)	All (*n* = 89)	*χ*^2^/*Z*	*P*
Demographics					
Sex				**1.30**	**0.254**
Male	28 (60.87%)	21 (48.84%)	49 (55.06%)		
Female	18 (39.13%)	22 (51.16%)	40 (44.94%)		
Age, year	58.50 (52.75, 64.50)	61 (56, 66)	60 (54.50, 66)	1.25	0.213
BMI, kg/m^2^	23.19 (20.75, 25.82)	22.59 (19.88, 24.65)	22.86 (20.50, 25.07)	1.42	0.154
Underlying conditions					
Hypertension	23 (50.00%)	17 (39.53%)	40 (44.94%)	0.98	0.321
Coronary artery disease	7 (15.22%)	2 (4.65%)	9 (10.11%)	–	0.159
Diabetes	9 (19.57%)	4 (9.30%)	13 (14.61%)	1.88	0.171
Tumor	0	0	0	–	1.000
Uremia	1 (2.17%)	0 (0.00%)	1 (1.12%)	–	1.000
COPD	0 (0.00%)	1 (2.33%)	1 (1.12%)	–	0.483
Cerebrovascular accident	2 (4.35%)	2 (4.65%)	4 (4.49%)	–	1.000
Vital signs upon admission					
SBP, mmHg	113.50 (102, 123.50)	117 (105, 124)	117 (102.50, 123.50)	0.45	0.654
DBP, mmHg	63 (56, 70)	67 (60, 70)	65 (58.50, 70)	1.16	0.244
Heart rate, bpm	85 (75, 96)	85 (76, 92)	85 (76, 93.50)	0.09	0.928
Oxygenation index	274 (194, 320.50)	312 (190, 372)	288 (191.50, 358)	1.24	0.214
BNP, pg/mL	620 (132.25, 2396)	400 (127, 1486)	565 (131, 1829.50)	0.84	0.400
Lactate, mmol/L	1.95 (1.10, 2.90)	1.70 (1.10, 3.30)	1.90 (1.10, 3.15)	0.02	0.980
SvO_2_, %	63.50 (57.75, 67)	63 (56, 71)	63 (56, 68)	0.02	0.984
CO_2_ gap, mmHg	10 (7.75, 12)	9 (6, 11)	10 (7, 11)	0.90	0.367
VIS score	8 (6, 15)	8 (6, 12)	8 (6, 13)	0.65	0.518

### Intraoperative clinical parameters

The median total surgical duration, CPB support time, and aortic cross-clamp time for the full cohort were 330 min, 139.50 min, and 91 min, respectively; the median intraoperative APACHE II score was 13. No statistically significant intergroup differences were identified in all intraoperative metrics (all *P* > 0.05), demonstrating comparable intraoperative management between study arms (Table 2).

**Table 2:** Patients’ intraoperative conditions between groups

Parameters	VExUS (*n* = 46)	Control (*n* = 43)	All (*n* = 89)	*χ*^2^/*Z*	*P*
Surgical duration, min	330 (275, 360)	320 (270, 420)	330 (270, 392.50)	0.08	0.934
support time, min	141 (107.75, 187.25)	138 (113.50, 193.50)	139.50 (112.50, 188.75)	0.27	0.786
Blocking time, min	92.50 (64.50, 111.25)	89 (73.50, 135)	91 (67, 114.50)	0.33	0.741
APACHE II	13 (9.75, 16)	12 (10, 16)	13 (10, 16)	0.17	0.863
Fluid balance	510 (75, 1300)	300 (-200, 1300)	450 (-25, 1300)	1.01	0.314

### Postoperative fluid, hemodynamic and transfusion metrics

Postoperative data on hemodynamics, fluid balance, and blood product transfusion were analyzed at 12 h, 24 h, and 48 h postoperatively. No significant differences were found in routine laboratory parameters, fluid intake, output, net balance between the two groups at each postoperative time point.The intergroup difference in albumin supplementation was statistically significant (12 h: *P* = 0.007; 24 h: *P* = 0.015). The control group had almost no albumin supplementation cases, with most values distribution at 0 g. At 48 h postoperatively, the VExUS-guided group displayed a more prominent negative net fluid balance, which is consistent with superior systemic fluid decongestion (Table 3).

**Table 3:** Postoperative serum, hemodynamic and fluid parameters at sequential time points

Parameters	VExUS (*n* = 46)	Control (*n* = 43)	All (*n* = 89)	*Z*	*P*
12-h					
SvO_2_, %	64 (57.75, 70)	66 (58, 72)	65 (58, 70.50)	0.51	0.610
CO_2_gap,mmHg	9 (7, 11)	8 (6, 10)	8 (7, 10.50)	1.15	0.249
VIS score	6 (4, 9)	7 (5, 10)	6 (4, 10)	0.78	0.437
Lactate, mmol/L	2.70 (1.40, 3.83)	2.20 (1.60, 4.40)	2.30 (1.40, 4)	0.30	0.761
Fluid intake, ml	1655 (1356.25, 2580)	1980 (1580, 2530)	1800 (1500, 2540)	1.73	0.083
Fluid output, ml	1670 (1316.25, 2691.25)	1775 (1230, 2510)	1720 (1265, 2535)	0.20	0.841
Fluid balance	95 (− 536, 595)	310 (− 170, 825)	220 (− 340, 765)	1.77	0.078
Red blood cell	0 (0, 2)	0 (0, 2)	0 (0, 2)	0.34	0.737
Plasma	0 (0, 400)	0 (0, 400)	0 (0, 400)	0.40	0.690
Albumin	0 (0, 20)	0 (0, 0)	0 (0, 0)	2.71	0.007
24-h					
SvO_2_, %	67 (60.75, 73)	69 (62, 75)	68 (62, 73)	0.67	0.500
CO_2_gap, mmHg	8 (5.75, 9)	7 (5, 9)	7 (5, 9)	0.52	0.603
VIS score	5 (3, 8.25)	5 (2.50, 8)	5 (3, 8)	0.63	0.528
Lactate, mmol/L	1.80 (1.18, 2.53)	1.60 (1.20, 2.60)	1.70 (1.20, 2.55)	0.12	0.908
Fluid intake, ml	1035 (807.50, 1342.50)	1070 (870, 1280)	1060 (815, 1292)	0.22	0.825
Fluid output, ml	1155 (927.50, 1442.50)	1190 (870, 1420)	1180 (915, 1430)	0.18	0.853
Fluid balance	− 152.50 (− 485, 215.50)	− 140 (− 360, 15)	− 140 (− 427.50, 52.50)	0.03	0.974
Red blood cell	0 (0, 0)	0 (0, 0)	0 (0, 0)	0.16	0.869
Plasma	0 (0, 0)	0 (0, 0)	0 (0, 0)	1.58	0.115
Albumin	0 (0, 0)	0 (0, 0)	0 (0, 0)	2.44	0.015
48-h					
Fluid intake, ml	1275 (1017.50, 1772)	1540 (1071.25, 1972.50)	1435 (1055, 1872.50)	1.08	0.278
Fluid output, ml	1862.50 (1302.50, 2567.50)	188 (1578.75, 2696.25)	1872.50 (1537.50, 2607.50)	0.17	0.866
Fluid balance	− 612.50 (− 957.50, − 227.50)	− 420 (− 937.50, − 77.50)	− 597 (− 952.50, − 156)	0.94	0.349

### Serial VExUS ultrasonographic metrics (intervention group only)

Table 4 presents the descriptive statistical results of VExUS-related indicators for the 46 patients in this group, with data summarized asmedian (IQR) and mean ± standard deviation.

**Table 4:** Serial VExUS ultrasound metrics in the intervention group

Parameters	Median (IQR)	Mean ± SD
0-h		
Interlobular vein	0 (0, 0)	0.39 ± 0.77
Interlobular renal artery resistive index	0.52 (0.50, 0.60)	0.54 ± 0.08
Inferior vena cava, mm	20 (16, 22)	18.87 ± 4.65
Main renal artery resistive index	0.64 (0.60, 0.69)	0.66 ± 0.08
Hepatic vein	0 (0, 2)	0.78 ± 0.92
Portal vein pulsatility index	0.23 (0.15, 0.39)	0.28 ± 0.17
VExUS grade	1 (0, 2)	1.02 ± 1.13
12-h		
Interlobular vein	0 (0, 1)	0.46 ± 0.78
Interlobular renal artery resistive index	0.56 (0.52, 0.62)	0.57 ± 0.07
Inferior vena cava, mm	21 (19, 23)	20.26 ± 3.81
Main renal artery resistive index	0.68 (0.62, 0.77)	0.69 ± 0.09
Hepatic vein	2 (0, 2)	1.28 ± 0.89
Portal vein	1 (0, 1)	0.83 ± 0.74
Portal vein pulsatility index	0.33 (0.25, 0.48)	0.35 ± 0.16
VExUS grade	2 (0, 2)	1.50 ± 1.09
24-h		
Interlobular vein	0 (0, 2)	0.65 ± 0.90
Interlobular renal artery resistive index	0.59 (0.50, 0.62)	0.58 ± 0.09
Inferior vena cava, mm	21 (17.75, 22)	20 ± 3.99
Main renal artery resistive index	0.70 (0.63, 0.78)	0.70 ± 0.10
Hepatic vein	2 (0, 2)	1.28 ± 0.91
Portal vein	1 (0, 2)	0.91 ± 0.84
Portal vein pulsatility index	0.33 (0.22, 0.51)	0.35 ± 0.17
VExUS grade	2 (0, 3)	1.59 ± 1.22

### Clinical outcomes

**Fig. 1:**
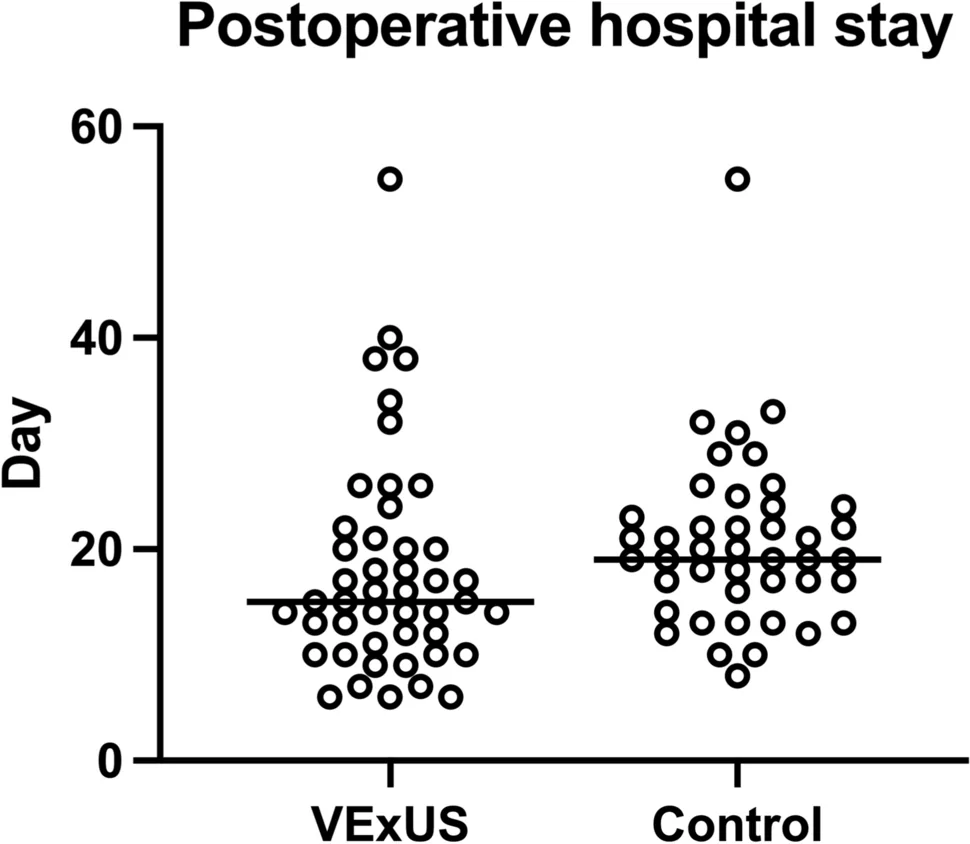
Postoperative hospital stay between the groups

Median ICU stay was 2 days, and the median postoperative hospital length of stay was 18 days; 97.75% of patients recovered uneventfully, with only 1 patient requiring subsequent hemodialysis. Intergroup outcome comparisons showed that median postoperative hospital stay in the VExUS-guided group was significantly shorter than that of the control group (15 days vs. 19 days, *P* = 0.027) (Table 5, Fig. 1).

**Table 5:** Patients’ clinical outcome

Parameters	VExUS (*n* = 46)	Control (*n* = 43)	All (*n* = 89)	*χ*^2^/*Z*	*P*
ICU stay, day	2 (2, 4)	3 (2, 5)	2 (2, 4.50)	1.03	0.302
Postoperative hospital stay, day	15 (10.75, 21.25)	19 (16, 24)	18 (13, 22.50)	2.21	0.027
Prognostic rehabilitation	44 (95.65%)	43 (100.00%)	87 (97.75%)	–	0.495
Hemodialysis	1 (2.22%)	0 (0.00%)	1 (1.15%)	–	1.000

## Discussion

This prospective randomized controlled study systematically evaluated the clinical value of VExUS-guided volume management in patients following CPB surgery. The key findings demonstrated that VExUS-guided volume management significantly shortened postoperative hospital stay and optimized early postoperative albumin compared with the conventional approach.

### Mechanisms linking VExUS guidance to reduced postoperative hospitalization

Patients undergoing CPB commonly develop systemic inflammatory response syndrome, capillary endothelial leakage, and third-space fluid sequestration, which predispose them to overt or subclinical systemic venous congestion. Balancing adequate tissue perfusion with timely decongestion represents the central therapeutic target of postoperative fluid care [10]. Multiple cohort studies have identified cumulative positive fluid balance within the first 24 postoperative hours as an independent adverse prognostic marker in cardiac surgery patients. Excess volume load elevates the risk of perioperative complication risk via three interconnected pathophysiological cascades:

First, excessive volume expansion damages vascular endothelial glycocalyx and impairs vascular barrier function, exacerbating interstitial capillary leakage [11, 12 ]; second, organ venous congestion, represented by intestinal edema destroys intestinal mucosal barrier, promotes endotoxin translocation and exacerbates systemic inflammatory response [13]; third, hemodilution caused by excessive fluid infusion reduces blood oxygen carrying capacity, which disturbs myocardial oxygen supply and demand balance in the high-risk postoperative stage [14].

Different from traditional single volume monitoring indicators such as CVP and IVC diameter which only reflect central circulatory preload, VExUS integrates multi-dimensional Doppler parameters of inferior vena cava, hepatic vein, portal vein and renal vein, which can quantitatively and comprehensively evaluate the congestion degree of systemic and important visceral venous circulation, and accurately identify occult venous congestion that cannot be detected by traditional indicators [15]. This facilitates earlier adjustment of fluid strategies, accelerating patient recovery. In this study, the VExUS-guided group initiated negative fluid balance interventions when the score exceeded 1, correcting potential venous congestion earlier than that in the control group, which may be associated with a lower incidence of venous congestion-related complications, such as pulmonary and intestinal edema, and a shorter postoperative hospital stay (15 days vs. 19 days, *P* = 0.027). This finding is consistent with the conclusions of Longino [16], which confirmed that VExUS-guided effectively identifies hidden venous congestion in critically ill patients, providing a basis for early intervention. Additionally, changes in portal vein blood flow parameters have been confirmed to be associated with severe complications such as congestive liver disease and encephalopathy after cardiac surgery [17–19], and they have predictive value for outcomes in patients with acute heart failure, further verifying their clinical value in volume management after CPB.

### Clinical significance of increased albumin administration

According to our study data, the median albumin infusion dose was 0 g in both groups at 12 and 24 h postoperatively. Nevertheless, the VExUS-guided group demonstrated wider data dispersion than the control group, and more patients in this group received high-dose albumin administration, resulting in statistically significant intergroup differences (*P* = 0.007 and *P* = 0.015, respectively).One key finding was that patients in the VExUS group received significantly greater albumin volumes at 12 and 24 h postoperatively, whereas vasoactive agent and diuretic use were comparable between the two cohorts. Patients undergoing cardiopulmonary bypass (CPB) frequently develop systemic inflammatory response syndrome (SIRS) and capillary leakage, which drive fluid extravasation into the third space. As outlined in the 202 4 review by Antit [20]and findings reported by Guinot [21], high VExUS grades—particularly increased portal vein pulsatility—predict congestive end-organ injury prior to derangements in serum laboratory markers. Diuretics were avoided in the present analysis, as these agents risk exacerbation of intravascular volume depletion; vasoactive medications were also withheld given their limited capacity to augment oncotic pressure. Instead, albumin was administered to increase colloid osmotic pressure, facilitate fluid mobilization back into the intravascular compartment, and preserve endothelial glycocalyx integrity. This management strategy was associated with shorter hospital stay (15 days vs. 19 days, *P* = 0.027).

### Fluid balance trends

Although there were no statistically significant differences in fluid balance between the two groups, the VExUS-guided group displayed a more obvious fluid negative balance at 48 h post-surgery (− 612.5 ml vs. − 420 ml), consistent with the principle of “early resuscitation, late restriction” in critical liquid management [22]. This trend suggests that the VExUS-guided approach can help clinicians precisely identify the timing of fluid “reduction or cessation,” avoiding the deleterious effects of excessive volume overload on organ function. However, the lack of statistical significance may be primarily due to the small sample size, which limits the statistical power, suggesting that large-sample studies are needed to validate the impact of the VExUS-guided approach on achieving a negative fluid balance.

### Study limitations

This study has the following limitations: First, as a single-center study with a limited sample size (89 patients in total), the results may be subject to selection biases, which restricts the generalizability of the conclusions. Second, insufficient statistical power from small cohort sizes precluded detection of significant differences for several clinically meaningful trends; for example, the pronounced negative fluid balance observed in the VExUS group at 48 h failed to reach statistical significance (*P* = 0.078), warranting replication in larger patient cohorts. Third, intraoperative allogeneic blood transfusion rates were numerically higher within the VExUS group (41.30% vs. 23.26%; *P* = 0.069), though this non-significant disparity was attributed to interindividual heterogeneity in baseline disease severity rather than the VExUS intervention, and did not alter the study’s primary and secondary outcome conclusions.

In this study, the intraoperative blood transfusion rate was higher in the VExUS-guided group (41.30%) than in the control group (23.26%), with an intergroup comparison *P* = 0.069, indicating no statistically significant difference. This trend was attributed to interindividual heterogeneity in baseline disease severity among enrolled patients rather than the grouped intervention, and it exerted no impact on the overall conclusions of the study.

## Conclusion

This study prospectively validated the clinical value of the VExUS-guided approach in volume management following CPB surgery. The VExUS-guided approach can optimize volume management strategies for patients following CPB surgery, shortening postoperative hospital stay and thus offering a potentially valuable tool for post-CPB volume management.

## Data Availability

The raw data supporting the findings of this study are available from the corresponding author upon reasonable request.

## References

[1] Arabi YM, Belley-Cote E, Carsetti A et al (2024) European Society of Intensive Care Medicine clinical practice guideline on fluid therapy in adult critically ill patients. Part 1: the choice of resuscitation fluids. Intensive Care Med 50(6):813–831 38771364 10.1007/s00134-024-07369-9

[2] Aya HD, Cecconi M, Hamilton M et al (2013) Goal-directed therapy in cardiac surgery: a systematic review and meta-analysis. Br J Anaesth 110(4):510–517 23447502 10.1093/bja/aet020

[3] Gao P, Liu J, Zhang P et al (2023) Goal-directed perfusion for reducing acute kidney injury in cardiac surgery: a systematic review and meta-analysis. Perfusion 38(3):591–599 35125028 10.1177/02676591211073783

[4] Boorsma EM, Ter Maaten JM, Damman K et al (2020) Congestion in heart failure: a contemporary look at physiology, diagnosis and treatment. Nat Rev Cardiol 17(10):641–655 32415147 10.1038/s41569-020-0379-7

[5] Vellinga NA, Ince C, Boerma EC (2013) Elevated central venous pressure is associated with impairment of microcirculatory blood flow in sepsis: a hypothesis generating post hoc analysis. BMC Anesthesiol 13:17 23919272 10.1186/1471-2253-13-17 PMC3750825

[6] Mekontso Dessap A, AlShamsi F, Belletti A et al (2025) European Society of Intensive Care Medicine (ESICM) 2025 clinical practice guideline on fluid therapy in adult critically ill patients: part 2—the volume of resuscitation fluids. Intensive Care Med 51(3):461–477 40163133 10.1007/s00134-025-07840-1

[7] Messina A, Matronola GM, Cecconi M (2025) Individualized fluid optimization and de-escalation in critically ill patients with septic shock. Curr Opin Crit Care 31(5):582–590 40762584 10.1097/MCC.0000000000001301

[8] Beaubien-Souligny W, Rola P, Haycock K et al (2020) Quantifying systemic congestion with point-of-care ultrasound: development of the venous excess ultrasound grading system. Ultrasound J 12(1):16 32270297 10.1186/s13089-020-00163-w PMC7142196

[9] Bhardwaj V, Vikneswaran G, Rola P et al (2020) Combination of inferior vena cava diameter, hepatic venous flow, and portal vein pulsatility index: venous excess ultrasound score (VEXUS score) in predicting acute kidney injury in patients with cardiorenal syndrome: a prospective cohort study. Indian J Crit Care Med 24(9):783–789 33132560 10.5005/jp-journals-10071-23570 PMC7584837

[10] Kontar L, Beaubien-Souligny W, Couture EJ et al (2023) Prolonged cardiovascular pharmacological support and fluid management after cardiac surgery. PLoS ONE. 10.1371/journal.pone.0285526 37167244 PMC10174538

[11] Rehm M, Bruegger D, Christ F et al (2007) Shedding of the endothelial glycocalyx in patients undergoing major vascular surgery with global and regional ischemia. Circulation 116(17):1896–1906 17923576 10.1161/CIRCULATIONAHA.106.684852

[12] Chappell D, Bruegger D, Potzel J et al (2014) Hypervolemia increases release of atrial natriuretic peptide and shedding of the endothelial glycocalyx. Crit Care (London, England) 18(5):538 10.1186/s13054-014-0538-5 PMC4201669 25497357

[13] Sundaram V, Fang JC (2016) Gastrointestinal and liver issues in heart failure. Circulation 133(17):1696–1703 27143152 10.1161/CIRCULATIONAHA.115.020894

[14] Kiel AM, Goodwill AG, Noblet JN et al (2017) Regulation of myocardial oxygen delivery in response to graded reductions in hematocrit: role of K(+) channels. Basic Res Cardiol 112(6):65 28965130 10.1007/s00395-017-0654-x PMC5938743

[15] Longino AA, Martin KC, Leyba KR et al (2024) Reliability and reproducibility of the venous excess ultrasound (VExUS) score, a multi-site prospective study: validating a novel ultrasound technique for comprehensive assessment of venous congestion. Crit Care (London, England) 28(1):197 10.1186/s13054-024-04961-9 PMC11165888 38858766

[16] Longino A, Martin K, Leyba K et al (2024) Prospective evaluation of venous excess ultrasound for estimation of venous congestion. Chest 165(3):590–600 37813180 10.1016/j.chest.2023.09.029 PMC11317813

[17] Eljaiek R, Cavayas YA, Rodrigue E et al (2019) High postoperative portal venous flow pulsatility indicates right ventricular dysfunction and predicts complications in cardiac surgery patients. Br J Anaesth 122(2):206–214 30686306 10.1016/j.bja.2018.09.028

[18] Benkreira A, Beaubien-Souligny W, Mailhot T et al (2019) Portal hypertension is associated with congestive encephalopathy and delirium after cardiac surgery. Can J Cardiol 35(9):1134–1141 31395469 10.1016/j.cjca.2019.04.006

[19] Styczynski G, Milewska A, Marczewska M et al (2016) Echocardiographic correlates of abnormal liver tests in patients with exacerbation of chronic heart failure. J Am Soc Echocardiogr 29(2):132–139 26549056 10.1016/j.echo.2015.09.012

[20] Pierre-Grégoire G (2025) VExUS score: optimizing its use in perioperative and critical care management. Crit Care (London, England) 29(1):472 10.1186/s13054-025-05687-y PMC12584479 41188936

[21] Antit S, Romdhane S, Mtiri I et al (2025) The role of venous excess ultrasound score in optimizing acute heart failure diagnosis and prognosis. J Saudi Heart Assoc 37(1):10 40297517 10.37616/2212-5043.1422 PMC12036827

[22] Ostermann M, Alshamsi F, Artigas Raventos A et al (2025) European Society of Intensive Care Medicine clinical practice guideline on fluid therapy in adult critically ill patients: part 3—fluid removal at de-escalation phase. Intensive Care Med 51(10):1749–1763 40828463 10.1007/s00134-025-08058-x

